# Multiple stages of tree seedling recruitment are altered in tropical forests degraded by selective logging

**DOI:** 10.1002/ece3.4352

**Published:** 2018-07-22

**Authors:** Rajeev Pillay, Fangyuan Hua, Bette A. Loiselle, Henry Bernard, Robert J. Fletcher

**Affiliations:** ^1^ Department of Wildlife Ecology and Conservation University of Florida Gainesville Florida USA; ^2^ Department of Zoology University of Cambridge Cambridge UK; ^3^ Center for Latin American Studies University of Florida Gainesville Florida USA; ^4^ Institute for Tropical Biology and Conservation Universiti Malaysia Sabah Kota Kinabalu Sabah Malaysia

**Keywords:** Borneo, *Dryobalanops lanceolata*, germination, mast‐fruiting, negative density‐dependence, seed predation, seed production, seedling survival, survival analysis

## Abstract

Tropical forest degradation is a global environmental issue. In degraded forests, seedling recruitment of canopy trees is vital for forest regeneration and recovery. We investigated how selective logging, a pervasive driver of tropical forest degradation, impacts canopy tree seedling recruitment, focusing on an endemic dipterocarp *Dryobalanops lanceolata* in Sabah, Borneo. During a mast‐fruiting event in intensively logged and nearby unlogged forest, we examined four stages of the seedling recruitment process: seed production, seed predation, and negative density‐dependent germination and seedling survival. Our results suggest that each stage of the seedling recruitment process is altered in logged forest. The seed crop of *D. lanceolata* trees in logged forest was one‐third smaller than that produced by trees in unlogged forest. The functional role of vertebrates in seed predation increased in logged forest while that of non‐vertebrates declined. Seeds in logged forest were less likely to germinate than those in unlogged forest. Germination increased with local‐scale conspecific seed density in unlogged forest, but seedling survival tended to decline. However, both germination and seedling survival increased with local‐scale conspecific seed density in logged forest. Notably, seed crop size, germination, and seedling survival tended to increase for larger trees in both unlogged and logged forests, suggesting that sustainable timber extraction and silvicultural practices designed to minimize damage to the residual stand are important to prevent seedling recruitment failure. Overall, these impacts sustained by several aspects of seedling recruitment in a mast‐fruiting year suggest that intensive selective logging may affect long‐term population dynamics of *D. lanceolata*. It is necessary to establish if other dipterocarp species, many of which are threatened by the timber trade, are similarly affected in tropical forests degraded by intensive selective logging.

## 1 INTRODUCTION

Vast areas of natural forest are being degraded around the world. In the tropics, selective logging characterized by unsustainable harvest rates and improper silvicultural practices is a major driver of forest degradation (Asner, Rudel, Aide, Defries, & Emerson, 2009; Martin, Newton, Pfeifer, Khoo, & Bullock, 2015; Putz et al., 2012). The consensus of recent insights into how selective logging (hereafter “logging”) affects biodiversity is that most species, across taxonomic groups, appear to persist in logged forests (Edwards, Tobias, Sheil, Meijaard, & Laurance, 2014; Putz et al., 2012; Wilcove, Giam, Edwards, Fisher, & Koh, 2013). What remains poorly understood, however, is how logging may affect numerous ecological processes that are the foundation of ecosystem functioning and maintenance (Ewers et al., 2015; Schleuning et al., 2011). Seedling recruitment is among the fundamental processes that determine the maintenance of plant diversity in forest ecosystems (Wright, 2002), and is a demographic bottleneck in plant population dynamics (Chambers & MacMahon, 1994; Chesson, 2000; Poulsen, Clark, & Bolker, 2012). In the aftermath of disturbances such as logging, seedling recruitment of canopy trees is vital for forest regeneration and recovery (Bagchi et al., 2011; Chazdon, 2003). Despite its importance, our knowledge of the impact of logging on seedling recruitment is limited (Bagchi et al., 2011; Curran et al., 1999).

There are several pathways by which logging may impact seedling recruitment. First, the removal of the largest and most reproductively active trees (Fisher et al., 2011) may directly reduce total seed production at the scale of the landscape (Bagchi et al., 2011). The remaining trees may also become spatially isolated from reproductive conspecifics, which may reduce cross‐pollination and, indirectly decrease seed crop size at the scale of individual trees (Ghazoul, Liston, & Boyle, 1998; Murawski, Nimal Gunatilleke, & Bawa, 1994). Second, the spatial isolation of adult trees from conspecifics in logged forests may concentrate seed predators at these trees (Bagchi et al., 2011), potentially increasing the strength of predator‐mediated negative density‐dependence (NDD), a process that underlies seedling recruitment beyond the seed production stage (Bagchi et al., 2014; Swamy & Terborgh, 2010). NDD, or the tendency for seeds and seedlings to exhibit reduced survival with increased conspecific density at local scales, is a pervasive demographic force that maintains plant community diversity by preventing the dominance of common species (Bagchi et al., 2014; Comita, Muller‐Landau, Aguilar, & Hubbell, 2010; Connell, 1971; Harms, Wright, Calderón, Hernández, & Herre, 2000; Janzen, 1970; Webb & Peart, 1999). Third, seed predator communities comprising insects, fungal pathogens, and vertebrates may undergo population shifts in logged forests (Ewers et al., 2015). The functional roles played by various seed predators may also change (Ewers et al., 2015), potentially altering the strength of NDD in constraining the recruitment of common species (Bagchi et al., 2011). Fourth, seeds in logged forests may be exposed to increased light penetration and hotter, drier air, and soil conditions due to the relatively open canopy (Hardwick et al., 2015). These abiotic factors can negatively affect seedling recruitment (Bruna, 1999). Thus, multiple stages of seedling recruitment may be altered in forests degraded by logging.

Understanding how logging may impact various stages of seedling recruitment is a particularly relevant issue in the dipterocarp‐dominated (Family Dipterocarpaceae) tropical rainforests of Southeast Asia. Not only is this ecosystem imperiled by logging (Asner et al., 2009), but its unique mast‐fruiting ecology also likely renders its seedling recruitment process to respond differently to logging compared with other tropical forest ecosystems (Bagchi et al., 2011). Mast‐fruiting in Southeast Asian rainforests occurs approximately every 3–9 years (Janzen, 1974) and is typically community wide: up to 88% of canopy tree species may fruit synchronously (Curran & Leighton, 2000; Curran et al., 1999). For dipterocarps, many species of which are targets of logging (Soepadmo, Saw, & Chung, 2002; Soepadmo & Wong, 1995), mast‐fruiting years account for the majority of seedling recruitment (Curran & Leighton, 2000; Janzen, 1974). Impacts on dipterocarp seedling recruitment during mast‐fruiting years may therefore have disproportionately important consequences for the recovery of logged forests in Southeast Asia (Bagchi et al., 2011; Curran & Leighton, 2000). While it is known that dipterocarps may undergo recruitment failure in logged forests of Southeast Asia during non‐mast years (Bagchi et al., 2011), empirical understanding of how various stages of seedling recruitment are impacted by logging during mast‐fruiting years is limited (Curran & Leighton, 2000; Curran & Webb, 2000).

Here, we investigate the impact of logging on the seedling recruitment process of an endemic and endangered dipterocarp species heavily targeted by logging, *Dryobalanops lanceolata* Burck (Ashton, 1998), during a mast‐fruiting event in Sabah, Borneo. We combined natural observations and an exclosure experiment over one mast‐fruiting season to test the following predictions. (1) Logging removes the largest, most reproductively active trees and may increase the spatial isolation of the remaining trees relative to each other. Therefore, we expected the seed crop would be smaller in logged forest than in unlogged forest. (2) Vertebrate and non‐vertebrate seed predator communities in logged forests may undergo population increases and declines, respectively (Ewers et al., 2015). Consequently, we expected the functional role of vertebrates in seed predation to increase and that of non‐vertebrates to decrease in logged forest. (3) Although the high seed densities typical of Southeast Asian mast‐fruiting forests usually reduce seed mortality by satiating predators (Janzen, 1970, 1974), we predicted the concentrated seed densities under spatially isolated trees in logged forests may allow NDD to operate (Bagchi et al., 2011). Therefore, we expected the relationship between seedling recruitment and local‐scale conspecific seed density would be stronger in logged forest than in unlogged forest. We focused on the seed‐to‐seedling transition phase over 3 months after seedfall, a demographic bottleneck that disproportionately influences the structure, dynamics, and composition of tree communities (Chambers & MacMahon, 1994; Chesson, 2000; Poulsen et al., 2012).

## 2 MATERIALS AND METHODS

### 2.1 Study area

We conducted fieldwork in two dipterocarp‐dominated forest sites spaced 65 km apart in Sabah, Malaysian Borneo. These sites are part of the experimental design of the Stability of Altered Forest Ecosystems (SAFE) Project (Ewers et al., 2011), and encompass primary forest within the Maliau Basin Conservation Area (MBCA) and repeatedly logged forest within the Kalabakan Forest Reserve (KFR), a logging concession in the Yayasan Sabah Forest Management Area. Overall, the logging gradient encompassed within the SAFE experimental design resembles the pattern of habitat conversion across the wider region (Ewers et al., 2011; Struebig et al., 2013).

The forest at MBCA (a 588.4 km^2^ protected area) represents the nearest topographically matched primary forest to the logged forest in KFR (Ewers et al., 2011; Struebig et al., 2013). The two sites are also similar in terms of soil nutrient profiles and physical properties (Riutta et al., 2018). The MBCA serves as the unlogged control site of the SAFE Project (Ewers et al., 2011). Two of the three control blocks at MBCA (hereafter “unlogged forest”) (OG1, OG2) have never been logged, while the third (OG3) was lightly logged in the 1970s and 1990s (see Ewers et al., 2011). As part of the SAFE Project, experimental forest fragments (1, 10, 100 ha) are being created in KFR (Ewers et al., 2011). Prior to this experiment, KFR was subjected to multiple rotations of logging, the first of which began in the 1970s (Chong, 2005; Fisher et al., 2011). Commercially valuable trees >60 cm diameter at breast height (DBH) were extracted and 112.96 m^3^/ha of timber was removed (Fisher et al., 2011). The second rotation, commencing in the 2000s (Chong, 2005; Fisher et al., 2011), encompassed three rounds during which trees >40 cm DBH were targeted (Fisher et al., 2011; Struebig et al., 2013). A volume of 25.87, 22.32, and 18.16 m^3^/ha of timber was extracted during each round, respectively (Yayasan Sabah, unpublished data). Logging ended in 2007–2008 (Fisher et al., 2011), by which time 179.31 m^3^/ha of timber was cumulatively removed (Struebig et al., 2013). Extensive collateral damage to forest structure also occurred due to the establishment of skid trails, access roads, and log‐landing areas (Wearn, Rowcliffe, Carbone, Bernard, & Ewers, 2013). Thus, the logging intensity was around 4.5 times the threshold of <40 m^3^/ha, beyond which residual stand damage surpasses the 25–30% limit considered sustainable (Martin et al., 2015). The six experimental blocks (A–F) at SAFE (hereafter “logged forest”) (Ewers et al., 2011) comprise a heterogeneous landscape that has been subjected to varying intensities and timings of timber extraction.

### 2.2 Study species

**Figure 1:**
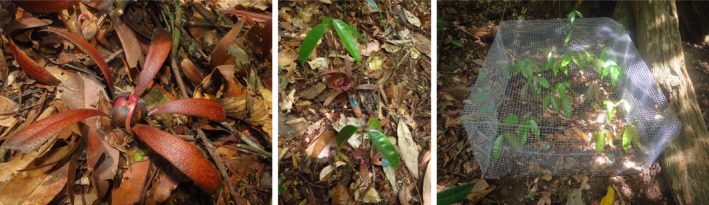
*Dryobalanops lanceolata* is an endangered dipterocarp endemic to Borneo. The seeds are wind dispersed and mostly fall near the parent tree. Left: A mature seed germinating. *D. lanceolata* seeds have five wings and are green when they fall from the tree but turn pink upon germination, within 5–7 days of dispersal. Center: Marked seedlings in a natural plot. Right: Seedlings in an experimental (vertebrate exclosure) plot. Photo credits: Rajeev Pillay

*Dryobalanops lanceolata* Burck is an emergent tree that can grow up to 80 m high (Soepadmo et al., 2002). Endemic to Borneo, it is widespread and common in the states of Sabah and Sarawak, growing in mixed‐dipterocarp forest on clay‐rich soils (Soepadmo & Wong, 1995; Soepadmo et al., 2002). The saplings are shade tolerant (Itoh, Yamakura, Ogino, & Lee, 1995) and can survive many years, expanding horizontally until a canopy gap opens up (Soepadmo et al., 2002). It is a hardwood species valued for its construction timber that is sold under the trade name *Kapur* (Soepadmo et al., 2002). Due to commercial harvesting and habitat loss, it is now rare outside protected areas and is classified as Endangered (IUCN Red List v. 2.3, Ashton, 1998). Like other dipterocarps, its winged seeds are dispersed by gyration (Figure 1), and mostly fall in close proximity to the crown of the parent tree (Itoh, Yamakura, Ogino, Lee, & Ashton, 1997). Its seeds and seedlings are attacked by fungal pathogens (*personal observation*), insects, and vertebrates such as bearded pigs (*Sus barbatus*) and various species of rodents (Itoh et al., 1995).

### 2.3 Study design

We conducted fieldwork during a mast‐fruiting event that occurred in 2014. In June and July, prior to the commencement of seedfall, we surveyed ~21.46 km of forest trails that passed through the diameters of the 1, 10, and 100 ha “fragments” of Blocks A, B, D, E, and F in the logged forest, and were able to locate only seven adult *D. lanceolata* trees in active fruiting condition (within Block E: two trees, within and around Block F: five trees). One of the trees in Block E was <40 m from the other reproductive conspecific. We removed this individual from this study to ensure non‐overlapping seed shadows, since prior work on *D. lanceolata* in Sarawak, Borneo (Itoh et al., 1997) indicates that all seeds are dispersed <40 m from the parent tree. We included the remaining six individuals in this study. Thus, the sparse number of adult conspecifics in active fruiting condition dictated our sample size at the level of individual trees. In the unlogged forest site, we surveyed approximately 6.5 km of forest trails each in Blocks OG1 and OG2 and located 15–20 (OG1) and 20–25 (OG2) adult *D. lanceolata* trees in active fruiting condition (we excluded OG3 from this study due to its logging history). The seed shadows of all trees in OG1 overlapped with those of at least one other conspecific adult. Therefore, we selected seven individuals in OG2 whose seed shadows did not overlap with those of other reproductive conspecifics. We considered each individual focal tree as an experimental unit, resulting in seven and six replicates at the level of individual trees in unlogged and logged forest, respectively. Individual trees are often used as experimental units in seed dispersal and seedling recruitment studies, and may be considered independent samples if their seed shadows do not overlap with those of other conspecifics (Bagchi et al., 2011; Holbrook & Loiselle, 2009; Poulsen et al., 2012).

The seven focal trees in unlogged forest were 303 m apart on average, with the smallest pairwise distance between two trees being 40 m. The six focal trees in logged forest were 1,690 m apart on average, with the smallest pairwise distance between two trees being 244 m, and cover considerable landscape heterogeneity in terms of logging intensity and time since logging (Wearn et al., 2013). On average, our focal trees were around 5.6 times further apart from each other in logged forest than in unlogged forest, which potentially indicates the intensity of extraction if *D. lanceolata* population densities were similar in both forest types prior to logging.

**Figure 2:**
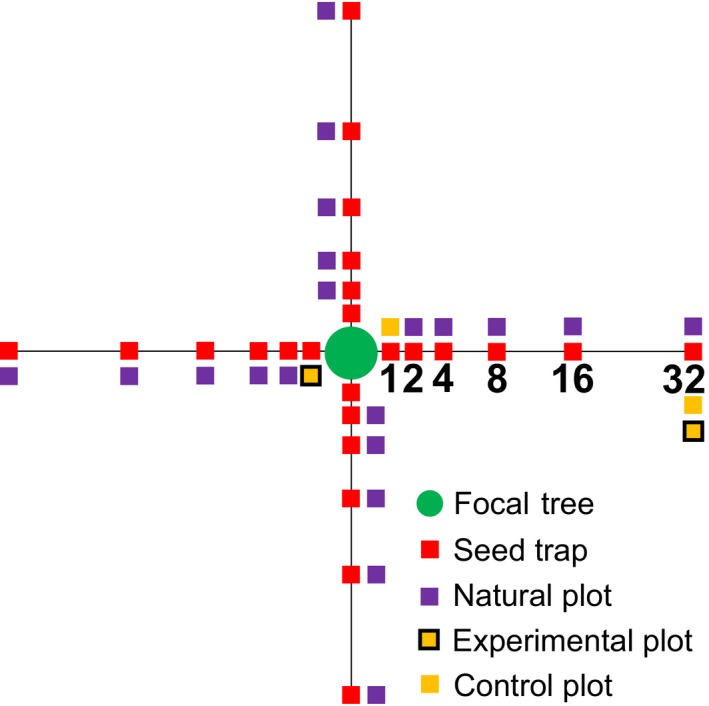
Study design showing seed traps (at 1, 2, 4, 8, 16, and 32 m distances), natural plots (at 2, 4, 8, 16, and 32 m distances) and paired experimental (vertebrate exclosures), and control plots (at 2 and 32 m distances) along four‐32 m transects from the base of focal *D. lanceolata* trees. Seed traps, natural and control plots were 1 × 1 m in area. Experimental plots were 1 × 1 × 0.5 m. Figure not to scale

We set up four 32 m transects extending from the base of each focal tree, with the first transect oriented along a random compass direction and each subsequent transect at 90° from the previous (Figure 2). We constrained transect length to 32 m because prior work indicated that few seeds reached this distance (Itoh et al., 1997). Along each transect, we used seed traps to quantify the seed crop and seed dispersal distance, setting them up soon after seedfall commenced in early August 2014. We deployed the seed traps on a log_2_ scale at 1, 2, 4, 8, 16, and 32 m distances (*n* = 24 seed traps/tree, Figure 2). Each trap was a 1 × 1 m nylon mesh net suspended by 1‐m tall PVC pipes at each corner. The validity of our data relies on the assumption that our focal trees had non‐overlapping seed shadows. Since 99.5% of all seeds fell into the seed traps within 16 m from their respective parent trees (see Section 2.1), a distance that is less than half of the minimum distance between any of our focal trees (i.e., 40 m), the seeds we included in this study should be predominantly, if not entirely, from their respective parent trees.

We monitored seed predation and density‐dependent patterns of germination and seedling survival of individual seeds that naturally dispersed into individual 1 × 1 m unmanipulated monitoring plots (hereafter “natural plots”). We thus utilized the conspecific seed/seedling density gradient naturally generated by the distance gradient for our assessment of NDD. We placed natural plots 2 m to the left of the seed traps along each transect, at 2, 4, 8, 16, and 32 m distances (*n* = 20 natural plots/tree, Figure 2). Additionally, around 1 month after the commencement of seedfall, we set up vertebrate‐exclosure plots (hereafter “experimental plots”) and paired, non‐exclosure plots (hereafter “control plots”) to test for the relative contribution of vertebrates versus insects and fungi to seed predation. We placed experimental and control plots at 2 and 32 m distances along one randomly selected transect at each focal tree (*n* = 2 plot pairs/tree, Figure 2). At 32 m, we placed each plot pair 2 m from each other and on the right side of the corresponding seed trap, randomly assigning the relative position of experimental versus control plots (i.e., which plot was placed adjacent to the corresponding seed trap). At 2 m, due to limited space near the base of the focal tree, we placed one plot on the right of the corresponding seed trap, and the paired plot at the corresponding position along the diagonally opposite transect, randomly assigning the relative position of experimental versus control plots (Figure 2). Due to logistical constraints, we omitted one of the most remote focal trees in logged forest in this comparison, and instead set up two additional plot pairs in the same way around another of our focal trees which was more accessible. Experimental plots were 1 × 1 × 0.5 m, with the exclosure made from steel wire of 1.27 × 1.27 cm mesh size to exclude all vertebrate seed predators but not insect seed predators or fungal pathogens (Figure 1). Control plots were open to all seed predators and pathogens.

For the paired experimental and control plots, we added *D. lanceolata* seeds at predefined densities ~1 month after commencement of seedfall, and thereafter continuously removed all conspecific seeds that fell into the control plots throughout the duration of the study. We used a seed density of 5 seeds/m^2^ for the paired plots at 2 m distances and 50 seeds/m^2^ for those at 32 m distances. For seeding the plots, we collected intact (i.e., no visible evidence of predator attack), mature seeds from around 20 non‐focal *D. lanceolata* trees in unlogged forest. We thoroughly mixed these seeds and placed them on the soil surface in a regular grid to mimic the natural seedfall pattern in which seeds gyrate from the parent trees and land on the ground. We thus distributed 770 seeds (*n* = 28 experimental and paired control plots) in each forest type. We note that this part of the study aims to provide only a supporting assessment of the relative importance of vertebrate and non‐vertebrate seed predators in unlogged and logged forests. We did not attempt to manipulate density and distance independently due to logistical difficulties in collecting and sowing additional seeds, a time‐sensitive undertaking because of the rapidity at which seeds germinate, to generate a spectrum of seed densities that would reflect scenarios under unlogged and logged conditions (cf. Bagchi et al., 2011; Poulsen et al., 2012).

### 2.4 Data collection

We collected seeds from the traps at two census intervals between 12 August and 4 November in logged forest, and between 1 September and 22 October in unlogged forest. We monitored the fate of every seed upon initiating the natural plots (at the same intervals as the seed traps), until the end of the study. We tagged all seeds with numbered plastic tags stapled to their wings (Figure 1) and continued tagging seeds that newly fell into the natural plots. At each census interval, we recorded the number of seeds that survived and died in each plot. For surviving seeds, we recorded if they were germinating and subsequently, whether they recruited into seedlings. We also scored each seed into one of the following categories (after Bagchi et al., 2011): intact (no visible signs of fungal, insect or vertebrate attack), germinated (defined as emergence of the radicle, Itoh et al., 1995), seedling (≥5 cm tall, expanded true leaves), vertebrate depredated (partially eaten, gnawed or removed), insect depredated (with entry/exit holes), fungus depredated (with fungal spores/mycelia). We note that the seeds falling into the natural plots would likely be exposed to both pre‐dispersal and postdispersal insect predators (Bagchi et al., 2011). We were careful to classify depredation status on the basis of the predator that we initially recorded to have attacked the seed/seedling (Lewis & Gripenberg, 2008). Thus, a seed with insect entry/exit holes at the initial census that was subsequently partially eaten or removed by vertebrates was scored as predated by insects. Due to the difficulty in ascertaining the initial predator between insects and fungal pathogens, we collapsed them into the non‐vertebrate category. We thus used two predator categories: vertebrate versus non‐vertebrate.

We measured a set of ecological covariates that can potentially influence various stages of seedling recruitment and thus should be accounted for in examining the impacts of logging. These covariates included three measures of tree size that are determinants of seed crop size and dispersal distance: DBH, height, and crown diameter (Cousens, Dytham, & Law, 2008; Norghauer, Nock, & Grogan, 2011). We also measured percentage canopy cover as a proxy for light availability, which in turn, may influence germination and seedling survival (Kobe, 1999). See Supporting information Appendix S1 for details on these measurements.

### 2.5 Statistical analyses

We first used principal‐components analysis (PCA) to identify major trends in tree size data and reduce the number of variables representing tree size. The first principal component (PC1) was positively correlated with all three variables representing tree size, had an eigenvalue >1, and explained 76.4% of the total variance in tree size data (Supporting information Table S1). We used the component scores generated from the loadings of PC1 as a surrogate for tree size in further statistical analyses.

We then used generalized linear mixed models (GLMMs) to analyze the impacts of logging on seed crop size and dispersal patterns, using the number of seeds in each seed trap as the response variable. We used forest type (unlogged vs. logged), distance from the focal tree, PC1 (hereafter “tree size”), and all two‐way interactions between forest type and the other variables as fixed effects. We included individual seed traps nested within trees as a random intercept in all models. We assumed a Poisson error distribution and a log link function. We tested for overdispersion by calculating the sum of squared Pearson residuals and comparing it to the residual degrees of freedom for each model (Zuur, Ieno, Walker, Saveliev, & Smith, 2009).

We also used GLMMs to analyze the impact of logging on the relative role of vertebrates versus non‐vertebrates in *D. lanceolata* seed and seedling predation. Our observations indicated that most seedlings died from predation (e.g., uprooted and partially/completely consumed/removed by vertebrates, cotyledons of germinated seedlings destroyed by insects, and/or fungal pathogens), rather than from leaf damage typically inflicted by herbivores (Coley & Barone, 1996). Therefore, we pooled data on seed and seedling predation. For the natural plots, we scored each seed/seedling as depredated by either vertebrate or non‐vertebrate predators and then modeled the probability of a seed/seedling being depredated by vertebrates (relative to non‐vertebrates) as a function of forest type as the fixed effect variable. For paired experimental and control plots, we counted the number of depredated seeds/seedlings out of the total number of seeds, and then modeled the probability of seed/seedling predation. We used forest type and treatment type (experimental vs. control plots) as fixed effect variables. In both analyses of seed/seedling predation, we included individual plots sequentially nested within transects and trees as a random intercept. We assumed a binomial error distribution and a logit link function. We note that nomadic herds of bearded pigs destroyed our experimental exclosures for four out of seven trees in unlogged forest (but not in logged forest) between 1 and 19 October (~30 days from the onset of seed/seedling monitoring in these plots). Therefore, for unlogged forest, we only considered data from the paired experimental and control plots for the three trees that were not depredated by bearded pigs (*n* = 330 seeds, 12 plots).

We used Cox regression mixed‐effects survival models to analyze the impact of logging on germination and seedling survival at the level of individual seeds and seedlings, with data collected from the natural plots. We first modeled seed survival until germination, with survival modeled as daily germination rate. Of the seeds that germinated, we subsequently modeled daily seedling survival until 3 months after seedfall. We incorporated seed/seedling age and used right‐censoring if a seed/seedling survived beyond the end of the monitoring period. For fixed effect variables, we included forest type, local‐scale (1 m^2^) conspecific seed/seedling density (hereafter “conspecific seed density”), distance from the parent tree and percentage canopy cover. We also included tree size and total conspecific seedfall at each tree during the study (surrogate for medium‐scale seed density) as fixed effect variables because these factors may vary between the two forest types, and potentially influence seedling recruitment patterns (Bagchi et al., 2011; Curran & Webb, 2000). Lastly, we included interactions between forest type and the five latter variables. For random effects, we sequentially nested the identities of plots within transects and trees to control for potential variation in microtopography that could cause differential germination and seedling survival (Born et al., 2015).

For the analyses described above, we built sets of sub‐models based on the global model, and compared models using Akaike's Information Criterion, adjusted for sample size (AIC_c_) (Burnham & Anderson, 2002). We considered models from the lowest AIC_c_ until the cumulative model weight exceeded 95%. We then used this 95% confidence model set as the basis for model averaging and statistical inference (Burnham & Anderson, 2002). We conducted all statistical analyses in R (v. 3.2.1) (R Development Core Team 2017) and used the *glmer* function in the package “lme4” (Bates, Mächler, Bolker, & Walker, 2015) to fit all GLMMs, the package “coxme” (Therneau, 2015) to fit survival models and the package “MuMIn” (Bartoń, 2016) for model selection and averaging.

## 3 RESULTS

### 3.1 Impact of logging on seed crop size

**Figure 3:**
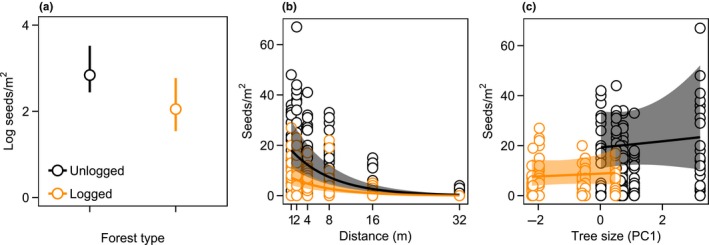
The relationship between *D. lanceolata* seed density and (a) forest type, (b) distance from focal trees in each forest type, and (c) tree size in each forest type. Error bars and the shaded regions of the curves are the predicted 95% confidence intervals

The five most parsimonious models, with a cumulative AICc weight of 100% (Supporting information Table S2), indicated that forest type, distance from the focal tree, tree size, and interactions between forest type and the other variables had support in explaining variation in *D. lanceolata* seed crop size and dispersal patterns. Consistent with prediction 1, the seed crop tended to be smaller in logged forest than in unlogged forest, even when accounting for tree size (Figure 3a, Table 1a). Over ~3 months (85 days), we collected 2,105 (*n* = 168 traps) and 718 (*n* = 144 traps) seeds in unlogged and logged forest, respectively. The average seed density (±1 *SE*) in unlogged forest was 12.52 (±0.99) seeds/m^2^ compared with 4.98 (±0.48) seeds/m^2^ in logged forest. The number of seeds declined with increasing distance from the focal trees in both forest types (Figure 3b, Table 1a). Consistent with patterns of local seed dispersal (Itoh et al., 1997), only 0.6% of all trapped seeds landed at 32 m in unlogged forest, and none in logged forest. Larger trees tended to produce more seeds in both forest types (Figure 3c, Table 1a), although trees in logged forest were smaller than those in unlogged forest (Supporting information Figure S1). We found no evidence for overdispersion (Supporting information Table S3).

**Table 1:** Model averaged estimates explaining variation in (a) seed crop size, (b) germination, and (c) seedling survival (up to 3 months after seedfall) of *D. lanceolata* in relation to the predictor variables that were supported by AICc‐based model selection criteria (Supporting information Tables S2, S6, S7)

Response and predictor variables	Estimate (*SE*)	95% CI
(a) Seed crop size		
Intercept	3.00 (0.29)	2.40–3.52
Logged forest	−0.80 (0.48)	−1.72–0.07
*Distance*	−*0.13 (0.01)*	−*0.14* to −*0.11*
Logged forest × distance	−0.01 (0.01)	−0.05–0.01
Tree size	0.06 (0.14)	−0.18–0.60
Logged forest × tree size	0.01 (0.10)	−0.54–0.80
(b) Germination		
*Logged forest*	*4.68 (3.62)*	*2.69–10.96*
*Conspecific seed density*	−*2.71 (2.38)*	−*7.44* to −*0.75*
*Logged forest* × *conspecific seed density*	*2.65 (2.35)*	*1.05–7.23*
Distance	0.03 (0.18)	−0.62–1.33
Logged forest × distance	−0.02 (0.16)	−1.34–0.91
Seedfall	−0.84 (1.57)	−6.02–1.04
Logged forest × seedfall	0.24 (1.49)	−4.22–14.28
Tree size	−0.18 (0.98)	−6.39–3.29
Logged forest × tree size	−0.14 (1.08)	−10.72–4.74
Canopy cover	0.01 (0.32)	−2.17–2.55
Logged forest × canopy cover	0.15 (0.84)	−1.55–7.75
(c) Seedling survival		
Logged forest	−7.10 (4.03)	−15.00–0.81
Conspecific seedling density	0.30 (0.15)	−0.01–0.60
*Logged forest* × *conspecific seedling density*	−*1.70 (0.43)*	−*2.55* to −*0.86*
*Distance*	*0.15 (0.09)*	*0.02–0.32*
*Logged forest* × *distance*	−*1.04 (0.67)*	−*2.31 to* −*0.12*
Seedfall	0.03 (0.58)	−1.19–1.26
Logged forest × seedfall	−2.03 (3.07)	−8.63–3.89
Tree size	−0.03 (0.96)	−2.06–1.99
*Logged forest* × *tree size*	−*3.16 (2.05)*	−*7.06 to* −*0.33*
Canopy cover	0.27 (0.28)	−0.24–0.87
Logged forest × canopy cover	−0.34 (1.10)	−2.71 to 1.91

Notes

Results for seed crop size represent coefficients (log‐scale) estimated with generalized linear mixed models. Results for germination and seedling survival represent log hazards estimated with Cox regression mixed‐effects survival models. Estimates in *italics* indicate that the 95% confidence intervals did not overlap zero, suggesting a strong effect of the predictor variable on the corresponding response variable.

### 3.2 Impact of logging on the predation of seeds and seedlings

**Figure 4:**
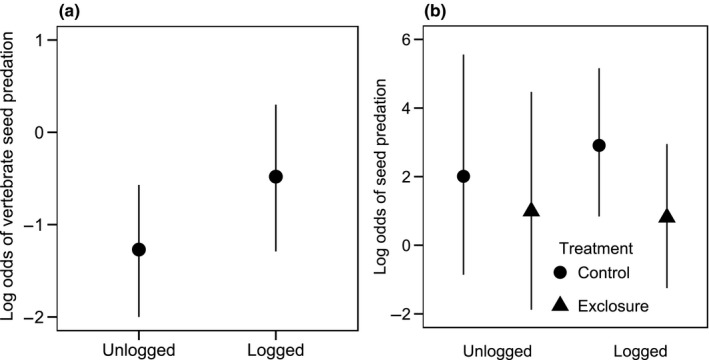
The odds of predation of *D. lanceolata* seeds by vertebrates, relative to non‐vertebrates, in (a) natural plots, and (b) experimental (vertebrate exclosures) and paired control plots in unlogged and logged forest. Error bars represent 95% confidence intervals

Over 3 months, we recorded an overall seed and seedling predation rate of 86.6 and 83.6% in unlogged (*n* = 1,423 seeds, 140 natural plots) and logged (*n* = 646 seeds, 120 natural plots) forest, respectively. A small number of seeds (unlogged forest: 5, logged forest: 8) died of desiccation. Consistent with prediction 2, there was a shift in the functional role of seed predators away from non‐vertebrates toward vertebrates in logged forest (Figure 4a,b). In the natural plots, the log odds of a seed/seedling being depredated by vertebrates (relative to non‐vertebrates) were higher in logged forest than in unlogged forest (β_logged_: 0.79, *SE*: 0.50, 95% CI: −0.28 to 1.85; Figure 4a; Supporting information Table S4). Further supporting prediction 2, the log odds of seed and seedling predation were lower in the experimental exclosure plots than in the control plots in logged forest (β_logged‐exclosure_: −2.10, *SE*: 0.57, 95% CI: −3.43 to −0.97; Figure 4b). In contrast, the effect of exclosure treatments on seed predation tended to be weaker in unlogged forest (β_unlogged‐exclosure_: −1.02, *SE*: 0.88, 95% CI: −2.90 to 0.89; Figure 4b; Supporting information Table S5).

### 3.3 Impact of logging on density‐dependent germination and seedling survival

**Figure 5:**
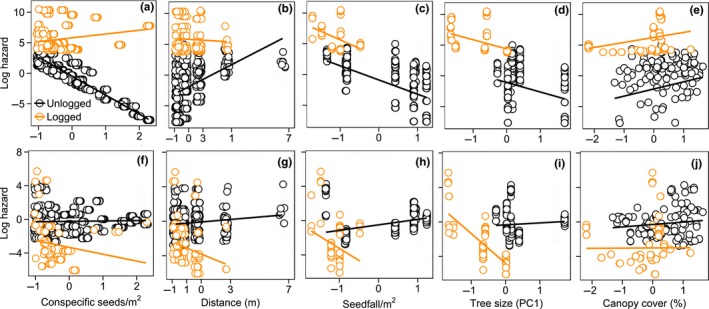
The relationships between *D. lanceolata* seed germination (top panel), seedling survival (bottom panel) and (a, f) conspecific seed/seedling density, (b, g) distance from the parent tree, (c, h) seedfall, (d, i) tree size, and (e, j) canopy cover in unlogged and logged forests

We monitored the germination and survival status of seeds that were alive in the natural plots at the first census (603 and 227 seeds in unlogged and logged forest, respectively) until the end of the study. The six most parsimonious models, with a cumulative AICc weight of 97% (Supporting information Table S6), indicated that forest type, conspecific seed density, distance from the parent tree, seedfall, tree size, canopy cover, and interactions between forest type and the other variables had support in explaining variation in seed germination. Seeds faced greater hazard (i.e., higher probability of mortality) in logged forest than in unlogged forest (Table 1b), as 47.1% of seeds failed to germinate in logged forest, compared with 0.8% in unlogged forest. Higher conspecific seed density was associated with lower seed mortality in unlogged forest, and this relationship also tended to apply in logged forest (Figure 5a, Table 1b). With increasing distance from parent trees, seed mortality tended to increase in both forest types, but this relationship was weaker in logged forest (Figure 5b, Table 1b). Higher seedfall and larger tree size tended to be associated with lower seed mortality in both forest types (Figure 5c,d; Table 1b). With increasing canopy cover, seed mortality tended to increase in both forest types (Figure 5e, Table 1b).

For explaining variation in seedling survival, the two most parsimonious models, with a cumulative AICc weight of 99% (Supporting information Table S7), indicated that forest type, conspecific seed density, distance from the parent tree, seedfall, tree size, canopy cover, and interactions between forest type and the other variables had support. Of the seeds that germinated, seedlings tended to have lower probability of mortality in logged forest than in unlogged forest (Table 1c), as 81.7% of germinated seedlings survived up to the end of the study in logged forest, compared with 31.1% in unlogged forest. Higher conspecific seedling density tended to be associated with higher seedling mortality in unlogged forest, but inconsistent with prediction 3, this relationship was reversed in logged forest (Figure 5f, Table 1c). With increasing distance from parent trees, seedling mortality increased in unlogged forest, but this relationship tended to be reversed in logged forest (Figure 5g, Table 1c). Higher seedfall tended to be associated with higher seedling mortality in unlogged forest, but this relationship also tended to be reversed in logged forest (Figure 5h, Table 1c). Like germination, larger tree size tended to be associated with lower seedling mortality in both forest types, and this relationship was stronger in logged forest (Figure 5i, Table 1c). With increasing canopy cover, seedling mortality tended to increase in unlogged forest, but this relationship tended to be reversed in logged forest (Figure 5j, Table 1c).

## 4 DISCUSSION

Our results demonstrate how multiple stages of the seedling recruitment process of a canopy tree species may be altered when tropical forests are degraded by intensive “selective” logging. Consistent with our predictions, the remaining reproductive trees in logged forest produced a markedly smaller seed crop than the trees in unlogged forest, even after accounting for tree size, and the functional role of vertebrates in seed predation increased in logged forest while that of non‐vertebrates declined. The strength of density‐dependence in dipterocarps may change from positive to negative at different life stages (Blundell & Peart, 2004; Curran & Webb, 2000). This appeared to be the case in unlogged forest where germination increased with local‐scale conspecific seed density, possibly because predators were satiated by the high mast‐fruiting seed densities, but seedling survival tended to decline (Figure 5a,f; Table 1b,c). However, inconsistent with our prediction of stronger NDD in logged forest, germination and especially seedling survival increased with local‐scale conspecific seed density, which suggests that logging may have affected predator‐mediated NDD during the seed‐to‐seedling transition bottleneck in *D. lanceolata*. Importantly, our findings applied to a mast‐fruiting year, when most dipterocarp recruitment occurs (Curran & Leighton, 2000; Janzen, 1974), suggesting that intensive logging may potentially affect long‐term population dynamics of *D. lanceolata*. Our findings add to knowledge of how ecological processes responsible for the maintenance of biodiversity may be affected by logging (Darrigo, dos Santos, & Venticinque, 2018; Ewers et al., 2015; Schleuning et al., 2011; Woodcock et al., 2013).

Increasing edaphic and drought stress in reproducing trees in logged forest may have contributed to the reduced seed crop (Curran et al., 1999; Hardwick et al., 2015). The consequences of seed limitation in logged forests for sapling and, ultimately, adult tree recruitment remain largely unexplored, but may be detrimental for long‐term tree population dynamics (Caughlin et al., 2015). Changes in the functional roles of non‐vertebrate seed predators relative to vertebrates in logged forest may be driven by numerical changes in predator abundance across groups (Ewers et al., 2015). The altered microclimatic conditions in logged forests are likely hostile to populations of insect seed predators and fungal pathogens (Ewers et al., 2015; Hardwick et al., 2015). Conversely, logged forests may provide increased resource availability for rodent seed predators and thus benefit their populations (Ewers et al., 2015).

The unique ecology of mast‐fruiting and the impact this phenomenon may have sustained in logged forest may explain the complex density‐dependent patterns of germination and seedling survival (Figure 5a,f). On the one hand, in unlogged forest, predator satiation may have been occurring at the high seed densities during the mast (Janzen, 1970), as potentially supported by our finding that germination tended to increase with higher seedfall (Figure 5c; Bagchi et al., 2011; Curran & Webb, 2000). Indeed, mast‐fruiting has been hypothesized to be an evolutionary response to density‐dependent mortality factors, allowing seeds to escape predation by satiating seed predators (Janzen, 1970, 1974). On the other hand, in logged forest, the concentrated seed densities under spatially isolated adult trees may allow NDD to operate (Bagchi et al., 2011; Curran & Webb, 2000). However, our findings of increased germination and especially seedling survival with higher conspecific seed density in logged forest (Figure 5a,f, Table 1b,c) were contrary to the expectations under NDD, and may be associated with the diminished functional role of non‐vertebrate seed predators in logged forest (Figure 4). NDD is primarily driven by host‐specific insect seed predators and fungal pathogens, which tend to concentrate among the high‐seed/seeding densities under the crowns of parent trees, thereby causing higher mortality where densities are higher (Bagchi et al., 2014; Connell, 1971; Janzen, 1970). These non‐vertebrate seed predators and pathogens may be declining in logged forests (Ewers et al., 2015), and their ecological role in maintaining NDD may be diminished. In contrast, the tendency of vertebrates to forage over large areas may render predation‐related mortality to be more spatially heterogeneous relative to seed/seedling densities (Swamy & Terborgh, 2010).

We note that seed crop size, germination, and seedling survival tended to increase for larger trees in both forest types (Figures 3c, 5d,i). This suggests that selective logging practices that harvest timber at sustainable rates and follow silvicultural practices designed to minimize damage to the residual stand (Asner et al., 2009; Martin et al., 2015; Putz et al., 2012) are critical to prevent seedling recruitment failure in dipterocarp forests. Seed germination and seedling survival of *D. lanceolata* tends to be positively associated with light availability (Itoh et al., 1995), which likely explains why seed and seedling mortality tended to increase with higher canopy cover (Figure 5e,j).

### 4.1 Caveats and limitations

Our study included seven and six *D. lanceolata* trees as independent experimental units in unlogged and logged forests, respectively (cf. Bagchi et al., 2011; Holbrook & Loiselle, 2009; Poulsen et al., 2012). The spatially clumped distributions of most adult fruiting individuals in unlogged forest and the few spatially scattered adult dipterocarps remaining in logged forest (of all species, *D. lanceolata* was the most abundant at seven individuals) precluded additional replication, at the level of individual trees, within forest type. The sparse number of adult dipterocarps in the logged forest is a reflection of the population reduction many timber tree species are subjected to after intensive selective logging (Martin et al., 2015).

The variable density of adult *D. lanceolata* trees between unlogged and logged forests may introduce confounding negative density‐dependent neighbourhood effects on seedling recruitment (Blundell & Peart, 2004; Stoll & Newbery, 2005). Previous research on the Dipterocarpaceae suggests that neighbourhood effects may occur when conspecific adults are within 20 m of each other (Stoll & Newbery, 2005). Our requirement of non‐overlapping seed shadows between focal trees ensured that trees were separated by at least 40 m. The relatively larger distance between the focal trees in our study, compared with the threshold distance of 20 m at which neighbourhood effects may occur (Stoll & Newbery, 2005), should thus serve to minimize this potential confounding factor.

In analyzing agents of seed and seedling predation, we did not distinguish between the different processes of seedling predation and herbivory. Of the seedlings that were alive in the natural plots at the end of the study, 0.17% and 1.76% showed signs of leaf damage typical of insect herbivory (Coley & Barone, 1996) in unlogged and logged forests, respectively. In the experimental and paired control plots, the percentage of seedlings with signs of leaf damage from herbivory was similarly small (0.61% and 0.65% in unlogged and logged forest, respectively). Thus, observed seedling herbivory rates were low. A longer‐term study may reveal altered seedling herbivory rates with logging (Darrigo et al., 2018), particularly because herbivorous insect biomass was found to increase with logging (Ewers et al., 2015). Our study examines the effects of intensive selective logging on the seed‐to‐seedling transition phase of a canopy dipterocarp. However, our findings may not represent the fate of seedlings over a longer time span because ecological changes caused by logging (e.g., herbivory) could continue to impact the survival of seedlings beyond the seed‐to‐seedling transition bottleneck (Caughlin et al., 2015).

We did not manipulate density and distance independently. Our use of seed density gradients naturally generated by the distance gradient can identify density‐dependent patterns of seedling recruitment (Harms et al., 2000; Webb & Peart, 1999). However, in the absence of experimental manipulation of density and distance effects independent of each other, our ability to understand the causes underlying observed patterns of density‐dependence in unlogged and logged forests is limited (Swamy & Terborgh, 2010). Future studies should leverage manipulative experiments for a nuanced understanding of the causes underlying altered patterns of seedling recruitment in the face of anthropogenic disturbances (Freckleton & Lewis, 2006).

## 5 CONCLUSION

We found that for *Dryobalanops lanceolata*, a dipterocarp species endangered by the commercial timber trade (Ashton, 1998), multiple stages of seedling recruitment were altered in selectively logged forest compared with unlogged forest during a mast‐fruiting year. Future studies should leverage broader logging gradients, additional species and experimental approaches over longer time frames (Bagchi et al., 2011; Martin et al., 2015; Riutta et al., 2018; Struebig et al., 2013; Swamy & Terborgh, 2010) to quantify the thresholds of logging intensity above which the ecological process of seedling recruitment may unravel. We highlight the importance of understanding the impacts of disturbances such as logging on ecological processes, which can complement numerical assessments of biodiversity to unmask impacts that may otherwise remain hidden.

## CONFLICT OF INTEREST

None declared.

## AUTHOR CONTRIBUTIONS

RP conceived this study; RP and RJF raised the grants for fieldwork; RP, BAL, RJF, and HB designed the methodology; RP collected the data and performed all statistical analyses; RP and FH led the writing of the manuscript. All authors contributed critically to the drafts and provided final approval for publication.

## DATA ACCESSIBILITY

Data can be accessed through the Dryad Digital Repository https://doi.org/10.5061/dryad.dk4t694 (Pillay, Hua, Loiselle, Bernard, & Fletcher, 2018).
