# Supplementary material for: Multiple stages of tree seedling recruitment are altered in tropical forests degraded by selective logging
Source: Ecol Evol. 2018 Jul 22;8(16):8231–42. doi: 10.1002/ece3.4352 (PMC6145000; doi:10.1002/ece3.4352)
Supplement: Supplementary file 1 [file ECE3-8-8231-s001.docx]

**SUPPORTING INFORMATION**

**Multiple stages of tree seedling recruitment are altered in tropical forests degraded by selective logging**

Rajeev Pillay, Fangyuan Hua, Bette A. Loiselle, Henry Bernard and Robert J. Fletcher, Jr.

**Appendix 1**

**Measurement of size attributes of focal trees and canopy cover**

We measured three important size attributes of focal trees that may determine seed crop size and seed dispersal distance: diameter at breast height (DBH), tree height, and tree crown diameter. We also measured percentage canopy cover as a proxy for light availability in each of the natural, experimental and control plots. Light availability is a vital determinant of germination and seedling survival (Kobe 1999). Varying levels of light availability in logged and unlogged forests may differentially influence seed germination and seedling survival.

1. **Diameter at breast height (DBH)**

We used a DBH tape (Forestry Suppliers Inc., Jackson, Mississippi, U.S.A.) to measure the DBH of each focal tree.

1. **Tree height**

We used a laser rangefinder (Nikon Prostaff 3; Nikon Corporation, Japan) to measure the height of each focal tree. The lead author positioned himself close to a focal tree such that the canopy was visible, and the sight of the laser rangefinder could be aimed near-vertically at the top of the canopy of the tree. Three height measurements were taken with the laser rangefinder, which were then averaged. The height of the author was added to the measured heights to obtain the height of each focal tree.

1. **Tree crown diameter**

We used a compass (Suunto KB-20, Suunto Oy, Finland) and a measuring tape to measure the crown diameter of each focal tree. We took two pairs of readings of crown radii from the base of each tree. For the first pair of readings, we first set up a line in a random compass direction from the base of a focal tree. A field assistant held the measuring tape at the base of the tree and the lead author moved along the designated compass direction until the edge of the crown was visible. This measurement constituted one radius of the crown. We then moved in the opposite direction and made a second measurement in a similar manner to complete the first pair of readings. Thereafter, we took a second pair of measurements in identical fashion, along a line oriented at 90° to the previous. We added each pair of crown radii measurements and then averaged both pairs to obtain the crown diameter of the tree.

1. **Canopy cover**

We used a type-A spherical densiometer to measure percentage canopy cover as a proxy for light availability (Lemmon 1956). We took four readings, one in each compass direction at each edge of each plot, and then averaged across the four readings. We took these readings at the edges of each plot, and not in the middle, to avoid stepping on the germinating seeds and seedlings within.

**Table S1.** Results of principal-components analysis (PCA) between diameter at breast height (DBH), height and crown diameter of individual focal trees. The first principal component (PC1) was positively correlated with all three variables representing tree size, had an eigenvalue > 1, and explained 76.4% of the total variance in tree size data. We used the component scores generated from the loadings of PC1 as a surrogate for tree size in further statistical analyses. All variables were centered and scaled prior to performing the PCA.

| Variable | Principal component | | |
| --- | --- | --- | --- |
|  | PC1 | PC2 | PC3 |
| Diameter at breast height DBH (cm) | 0.63 | -0.04 | 0.76 |
| Tree height (m) | 0.53 | 0.74 | -0.40 |
| Tree crown diameter (m) | 0.55 | -0.66 | -0.49 |
|  |  |  |  |
| *Importance of each component* |  |  |  |
| Standard deviation | 1.51 | 0.77 | 0.33 |
| Eigenvalue | 2.29 | 0.60 | 0.11 |
| % variance explained | 76.40 | 19.98 | 3.61 |

**Figure S1.** Size measurements of focal trees (Unlogged forest: n = 7, Logged forest: n = 6). On average, focal trees in unlogged forest were larger than those in logged forest in terms of diameter at breast height (DBH), height and crown diameter. Error bars represent ±1 SE.


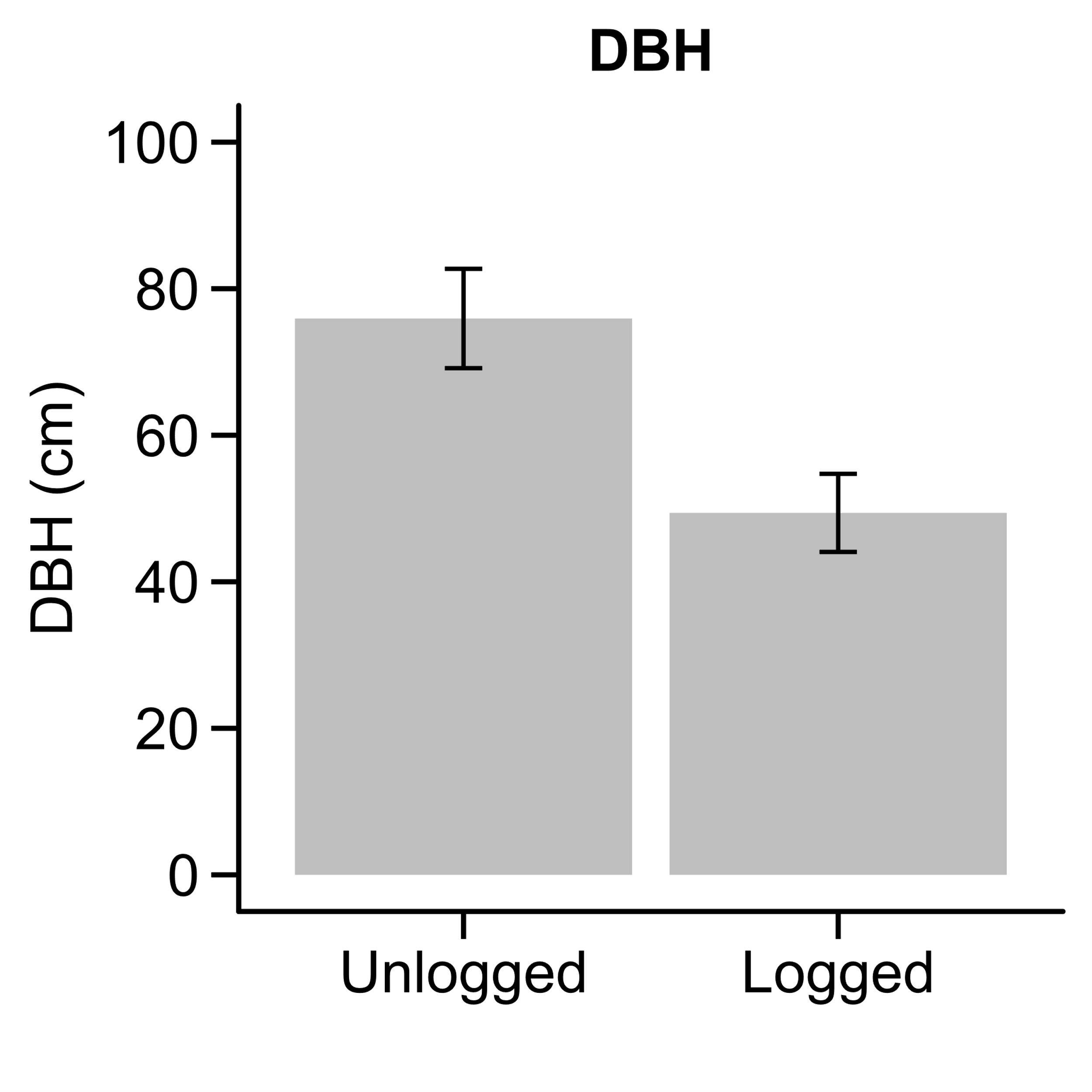

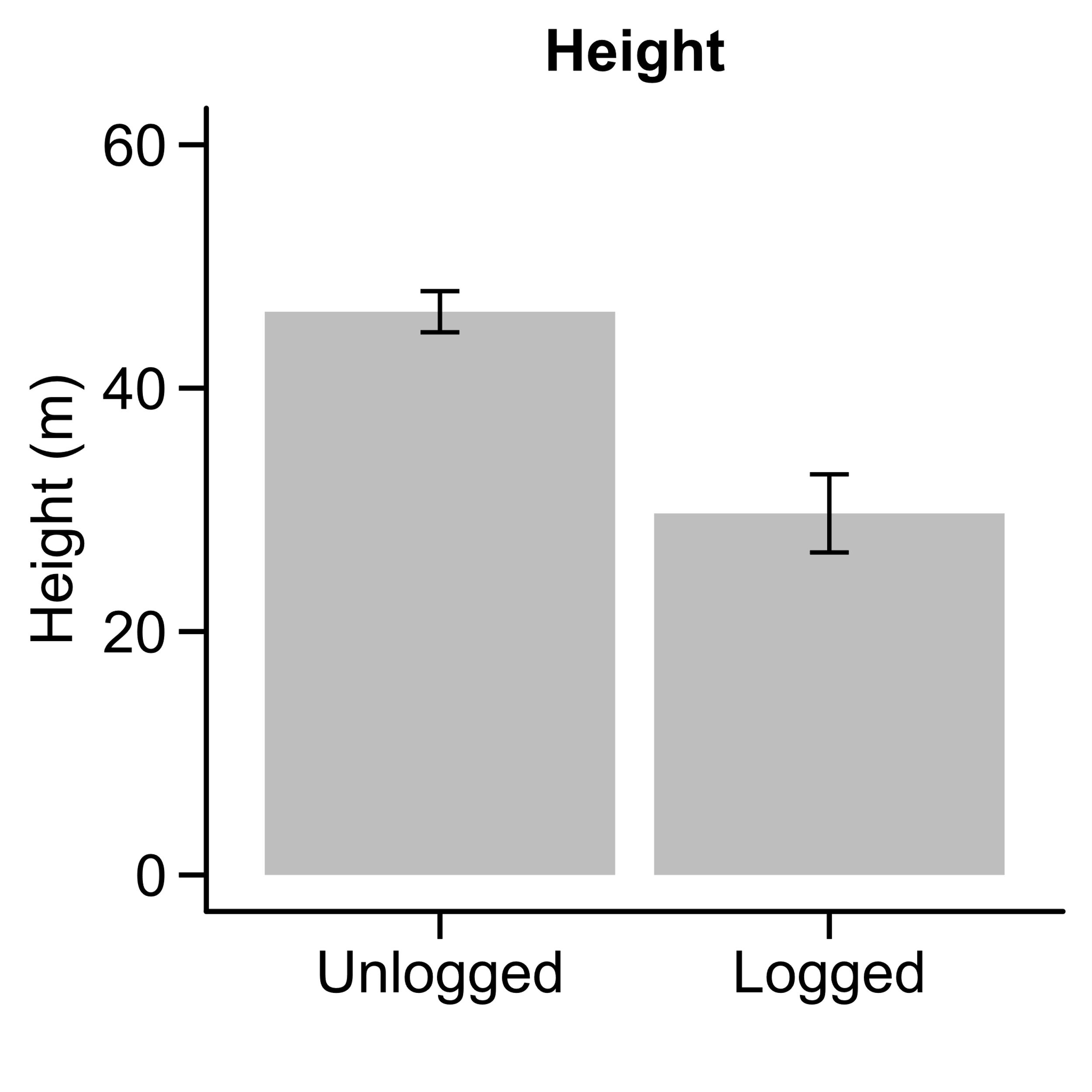

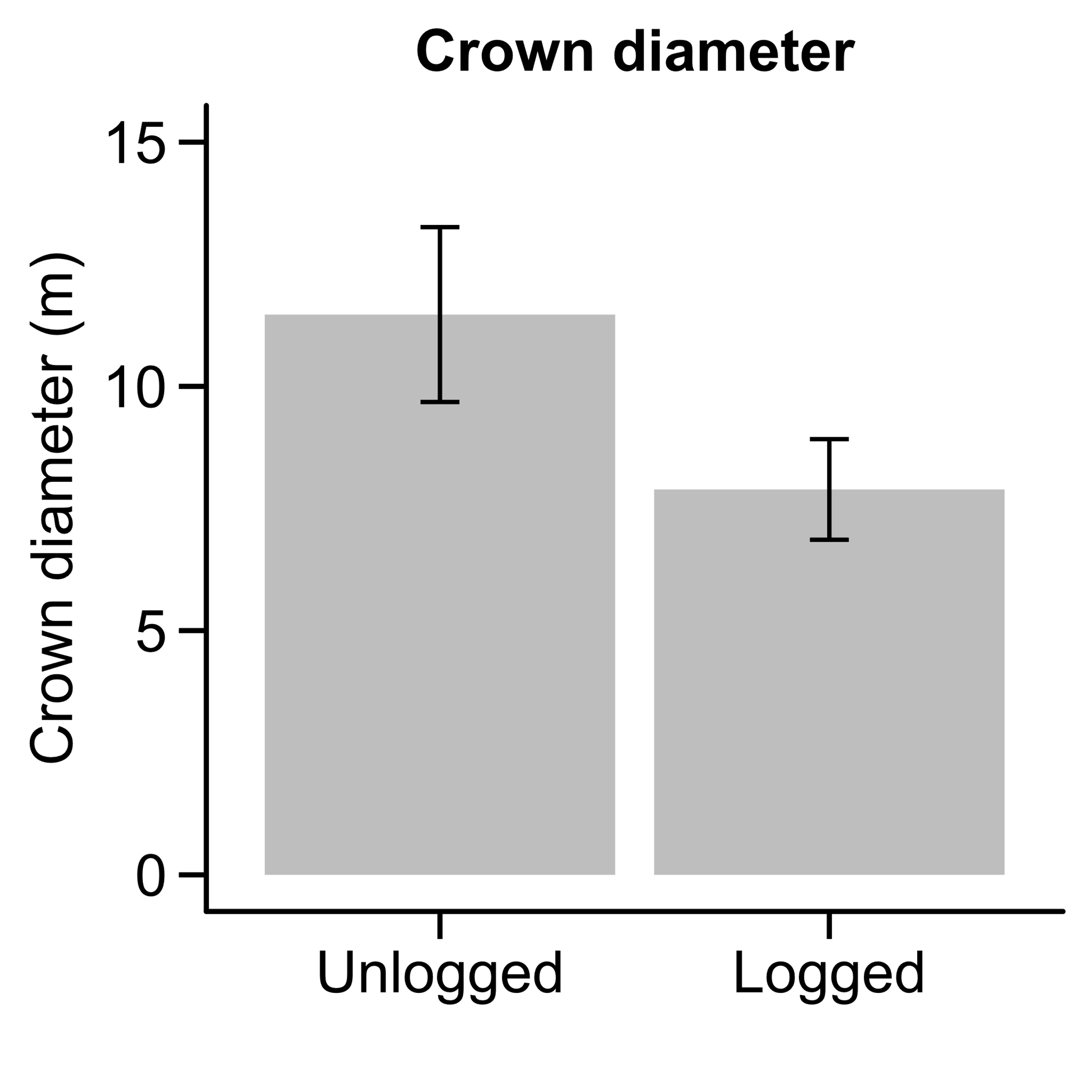


**Table S2.** Model selection results from generalized linear mixed models testing for variation in seed crop size as a function of (i) forest type (ftype: unlogged versus logged), (ii) distance from the focal tree, (iii) tree size (eigenvectors of PC1 from principal-components analysis, Table S1), and (iv) all two-way interactions between forest type and the other variables. We considered individual seed traps nested within individual focal trees as a random effect in all models. Following model selection, the top five models (*in italics*), which cumulatively had a Akaike model weight of 100%, were used to generate the model averaged estimates in Table 1a. We found no evidence for overdispersion (Table S3).

| Model | K | df | AICc | ∆AICc | AICc Wt. | log likelihood |
| --- | --- | --- | --- | --- | --- | --- |
| *ftype + distance + (1\|tree/trap)* | *5* | *307* | *1662.49* | *0.00* | *0.38* | *-826.15* |
| *ftype + tree size + distance + (1\|tree/trap)* | *6* | *306* | *1663.26* | *0.78* | *0.26* | *-825.49* |
| *ftype + distance + ftype* × distance *+ (1\|tree/trap)* | *6* | *306* | *1663.32* | *0.83* | *0.25* | *-825.52* |
| *ftype + tree size + distance + ftype* × *tree size + ftype* × *distance + (1\|tree/trap)* | *8* | *304* | *1666.09* | *3.61* | *0.06* | *-824.81* |
| *distance + (1\|tree/trap)* | *4* | *308* | *1666.63* | *4.15* | *0.05* | *-829.25* |
| tree size + (1\|tree/trap) | 4 | 308 | 1994.33 | 331.85 | 0.00 | -993.10 |
| ftype + (1\|tree/trap) | 4 | 308 | 1994.82 | 332.33 | 0.00 | -993.34 |
| ftype + tree size + (1\|tree/trap) | 5 | 307 | 1995.44 | 332.95 | 0.00 | -992.62 |
| ftype + tree size + ftype × tree size + (1\|tree/trap) | 6 | 306 | 1997.15 | 334.67 | 0.00 | -992.44 |
| 1 + (1\|tree/trap) | 3 | 309 | 1998.43 | 335.94 | 0.00 | -996.18 |

**Notes** **–** K: number of parameters in a model; df: degrees of freedom of a model; AICc: Akaike’s Information Criterion, corrected for sample size; ΔAICc: AICc for model *i* -minAICc; AICc Wt.: AICc model weight.

**Table S3.** Pearson’s residuals for Poisson generalized linear mixed models testing for variation in seed crop size as a function of (i) forest type (ftype: unlogged vs. logged), (ii) distance from the focal tree and, (iii) tree size. See Table S2 for model selection results. If Pearson’s residuals > 1, then the model is overdispersed (Zuur *et al.* 2009).

| Model | Pearson’s Residuals |
| --- | --- |
| ftype + distance + (1\|tree/trap) | 0.56 |
| ftype + tree size + distance + (1\|tree/trap) | 0.56 |
| ftype + distance + ftype × distance + (1\|tree/trap) | 0.56 |
| ftype + tree size + distance + ftype × tree size + ftype × distance + (1\|tree/trap) | 0.57 |
| distance + (1\|tree/trap) | 0.56 |
| tree size + (1\|tree/trap) | 0.45 |
| ftype + (1\|tree/trap) | 0.45 |
| ftype + tree size + (1\|tree/trap) | 0.45 |
| ftype + tree size + ftype × tree size + (1\|tree/trap) | 0.45 |
| 1 + (1\|tree/trap) | 0.45 |

**Table S4.** Model selection results from generalized linear mixed models testing for variation in seed and seedling predation by vertebrates versus non-vertebrates in unlogged and logged forests (ftype). We considered individual natural plots sequentially nested within transects and focal trees as a random effect in all models. We used the top model to generate the estimates shown in Figure 4a.

| model | K | df | AICc | ∆AICc | AICc Wt. | log likelihood |
| --- | --- | --- | --- | --- | --- | --- |
| ftype + (1\|tree/transect/plot) | 5 | 1767 | 2023.59 | 0 | 0.53 | -1006.78 |
| 1 + (1\|tree/transect/plot) | 4 | 1768 | 2023.80 | 0.22 | 0.47 | -1007.89 |

**Notes –** K: Number of parameters in a model; df: degrees of freedom of a model; AICc: Akaike’s Information Criterion, corrected for sample size; ΔAICc: AICc for model *i* -minAICc; AICc Wt.: AICc model weight.

**Table S5.** Model selection results from generalized linear mixed models testing for variation in seed and seedling predation in experimental (vertebrate exclosures) and paired control (non-exclosure) plots in unlogged and logged forest. We considered individual plots sequentially nested within transects and focal trees as a random effect in both models. We used the top model to generate the estimates shown in Figure 4b.

| Model | K | df | AICc | ∆AICc | AICc Wt. | log likelihood |
| --- | --- | --- | --- | --- | --- | --- |
| treatment + (1\|tree/transect/plot) | 7 | 1093 | 936.29 | 0 | 0.96 | -461.09 |
| 1 + (1\|tree/transect/plot) | 4 | 1096 | 942.42 | 6.13 | 0.04 | -467.19 |

**Notes –** K: Number of parameters in a model; df: degrees of freedom of a model; AICc: Akaike’s Information Criterion, corrected for sample size; ΔAICc: AICc for model *i* -minAICc; AICc Wt.: AICc model weight. *Fixed effect –* treatment was coded as unlogged forest-exclosure, unlogged forest-control, logged forest-exclosure and logged forest-control.

**Table S6.** Model selection results from Cox regression mixed-effects survival models testing for variation in seed germination as a function of (i) forest type (ftype: unlogged versus logged), (ii) local-scale conspecific seed density (consp), (iii) distance from the focal tree (dist), (iv) canopy cover (cc), (v) tree size (eigenvectors of PC1 from principal-components analysis, Table S1), (vi) total conspecific seedfall at each tree (avgseeds) and, (vii) all two-way interactions between forest type and the other variables. We considered individual natural plots sequentially nested within transects and focal trees as a random effect in all models. Following model selection, the top six models (*in italics*), which cumulatively had a Akaike model weight of 97%, were used to generate the model averaged estimates in Table 1b and the predicted lines shown in Figure 5 (top panel).

| Model | K | Adjusted df | AICc | ∆AICc | AICc Wt. | log likelihood |
| --- | --- | --- | --- | --- | --- | --- |
| *ftype + consp + ftype* × *consp + (1\|tree/transect/plot)* | *6* | *25.91* | *842.17* | *0* | *0.57* | *-394.31* |
| *avgseeds + (1\|tree/transect/plot)* | *4* | *22.07* | *843.76* | *1.59* | *0.26* | *-399.18* |
| *ftype + cc + consp + dist + tree size + avgseeds + ftype* × *cc + ftype* × *consp + ftype* × *dist + ftype* × *tree size + ftype* ×  *avgseeds + (1\|tree/transect/plot)* | *14* | *28.49* | *847.19* | *5.02* | *0.05* | *-394.06* |
| *tree size + (1\|tree/transect/plot)* | *4* | *23.29* | *847.22* | *5.05* | *0.05* | *-399.62* |
| *ftype + dist + ftype* × *dist + (1\|tree/transect/plot)* | *6* | *26.1* | *848.58* | *6.41* | *0.02* | *-397.31* |
| *ftype + cc + consp + dist + tree size + avgseeds + (1\|tree/transect/plot)* | *9* | *25.2* | *848.78* | *6.61* | *0.02* | *-398.37* |
| ftype + avgseeds + ftype × avgseeds + (1\|tree/transect/plot) | 6 | 23.65 | 849.61 | 7.44 | 0.01 | -400.43 |
| ftype + (1\|tree/transect/plot) | 4 | 24.6 | 850.68 | 8.51 | 0.01 | -399.96 |
| ftype + tree size + ftype × tree size + (1\|tree/transect/plot) | 6 | 24.05 | 851.079 | 8.91 | 0.01 | -400.74 |
| ftype + cc + ftype × cc + (1\|tree/transect/plot) | 6 | 25.74 | 853.49 | 11.32 | 0.002 | -400.14 |
| dist + (1\|tree/transect/plot) | 4 | 31.28 | 855.43 | 13.26 | 0.002 | -395.17 |
| cc + (1\|tree/transect/plot) | 4 | 17.54 | 881.81 | 39.64 | 0.000 | -422.96 |
| consp + (1\|tree/transect/plot) | 4 | 16.26 | 890.80 | 48.63 | 0.000 | -428.80 |

**Notes –** K: Number of parameters in a model; Adjusted df: degrees of freedom of a model penalized for random effects (Therneau 2018); AICc: Akaike’s Information Criterion, corrected for sample size; ΔAICc: AICc for model *i* -minAICc; AICc Wt.: AICc model weight. All predictor variables were centered and scaled prior to analyses.

**Table S7.** Model selection results from Cox regression mixed-effects survival models testing for variation in seedling survival as a function of (i) forest type (ftype: unlogged versus logged), (ii) local-scale conspecific seed density (consp), (iii) distance from the focal tree (dist), (iv) canopy cover (cc), (v) tree size (eigenvectors of PC1 from principal-components analysis, Table S1), (vi) total conspecific seedfall at each tree (avgseeds) and, (vii) all two-way interactions between forest type and the other variables. We considered individual natural plots sequentially nested within transects and focal trees as a random effect in all models. Following model selection, the top two models (*in italics*)*,* which cumulatively had a AICc model weight of 99%, were used to generate the model averaged estimates in Table 1c and the predicted lines shown in Figure 5 (bottom panel).

| Model | K | Adjusted df | AICc | ∆AICc | AICc Wt. | log likelihood |
| --- | --- | --- | --- | --- | --- | --- |
| *ftype + cc + consp + dist + tree size + avgseeds + ftype* × *cc + ftype* × *consp + ftype* × *dist + ftype* × *tree size + ftype* × *avgseeds + (1\|tree/transect/plot)* | *14* | *76.98* | *4838.68* | *0* | *0.85* | *-2332.98* |
| *ftype + consp + ftype* × *consp + (1\|tree/transect/plot)* | *6* | *75.71* | *4842.23* | *3.55* | *0.14* | *-2336.35* |
| ftype + tree size + ftype × tree size + (1\|tree/transect/plot) | 6 | 72.46 | 4854.26 | 15.57 | 0.0003 | -2346.41 |
| cc + (1\|tree/transect/plot) | 4 | 71.96 | 4855.64 | 16.96 | 0.0002 | -2347.72 |
| ftype + cc + consp + dist + tree size + avgseeds + (1\|tree/transect/plot) | 9 | 73.42 | 4855.83 | 17.15 | 0.0002 | -2346.00 |
| ftype + (1\|tree/transect/plot) | 4 | 72.47 | 4855.99 | 17.31 | 0.0001 | -2347.26 |
| ftype + avgseeds + ftype × avgseeds + (1\|tree/transect/plot) | 6 | 72.64 | 4856.06 | 17.38 | 0.0001 | -2347.09 |
| dist + (1\|tree/transect/plot) | 4 | 73.04 | 4856.98 | 18.29 | 0.0000 | -2347.05 |
| ftype + cc + ftype × cc + (1\|tree/transect/plot) | 6 | 72.24 | 4857.04 | 18.35 | 0.0000 | -2348.07 |
| tree size + (1\|tree/transect/plot) | 4 | 72.38 | 4857.10 | 18.41 | 0.0000 | -2347.93 |
| avgseeds + (1\|tree/transect/plot) | 4 | 71.94 | 4857.13 | 18.45 | 0.0000 | -2348.49 |
| consp + (1\|tree/transect/plot) | 4 | 73.16 | 4857.85 | 19.17 | 0.0000 | -2347.34 |
| ftype + dist + ftype × dist + (1\|tree/transect/plot) | 6 | 73.39 | 4858.08 | 19.40 | 0.0000 | -2347.17 |

**Notes –** K: Number of parameters in a model; Adjusted df: degrees of freedom of a model penalized for random effects (Therneau 2018); AICc: Akaike’s Information Criterion, corrected for sample size; ΔAICc: AICc for model *i* -minAICc; AICc Wt.: AICc model weight. All predictor variables were centered and scaled prior to analyses.

# **References**

Kobe, R.K. (1999) Light gradient partitioning among tropical tree species through seedling mortality & growth. *Ecology*, **80**, 187–201.

Lemmon, P.E. (1956) A spherical densiometer for estimating forest overstory density. *Forest Science*, **2**, 314–320.

Therneau, T. (2018) *Mixed Effects Cox Models*. Mayo Clinic.

Zuur, A.F., Ieno, E.N., Walker, N.J., Saveliev, A.A. & Smith, G.M. (2009) *Mixed Effects Models and Extensions in Ecology with R*. Springer, New York.
